# Application and Utility of Continuous Glucose Monitoring in Pregnancy: A Systematic Review

**DOI:** 10.3389/fendo.2019.00697

**Published:** 2019-10-11

**Authors:** Qi Yu, Izzuddin M. Aris, Kok Hian Tan, Ling-Jun Li

**Affiliations:** ^1^Duke Medical School, Duke University, Durham, NC, United States; ^2^Division of Chronic Disease Research Across the Lifecourse, Department of Population Medicine, Harvard Medical School and Harvard Pilgrim Health Care Institute, Boston, MA, United States; ^3^Division of O&G, KK Women's and Children's Hospital, Singapore, Singapore; ^4^OBGYN ACP, Duke-NUS Medical School, Singapore, Singapore

**Keywords:** gestational diabetes, continuous glucose monitoring, self-monitoring of blood glucose, literature review, maternal and fetal outcomes

## Abstract

**Background:** In the past decade, continuous glucose monitoring (CGM) has been proven to have similar accuracy to self-monitoring of blood glucose (SMBG) and yet provides better therapy optimization and detects trends in glucose values due to higher frequency of testing. Even though the feasibility and utility of CGM has been proven successfully in Type 1 and 2 diabetes, there is a lack of knowledge of its application and effectiveness in pregnancy, especially in gestational diabetes mellitus (GDM). In this review, we aimed to summarize and evaluate the updated scientific evidence on the application of CGM in pregnancies complicated with GDM.

**Methods:** A search using keywords related to CGM and GDM on PubMed was conducted and articles were filtered based on full text, year of publication (Jan 1998–Dec 2018), human subject studies, and written in English. Reviews and duplicate articles were removed. A final total of 29 articles were included in this review.

**Results:** In terms of maternal and fetal outcomes, inconsistent evidence was reported. Among GDM patients using CGM and SMBG, two randomized controlled trials (RCTs) found no significant differences in macrosomia, birth weight (BW), and gestational age (GA) at delivery between these two groups, while one prospective cohort found a lower incidence of cesarean section and macrosomia in CGM use subjects. Furthermore, CGM use was consistently found to have increased detection in dysglycemia and glycemic variability compared to SMBG. In terms of clinical utility, CGM use led to more treatment adjustments and lower gestational weight gain (GWG). Lastly, CGM use showed higher postprandial glucose levels in GDM-complicated pregnancies than in normal pregnancies.

**Conclusion:** Current updated evidence suggests that CGM is superior to SMBG among GDM pregnancies in terms of detecting hypoglycemic and hyperglycemic episodes, which might result in an improvement of maternal and fetal outcomes. In addition, CGM detects a wider glycemic variability in GDM mothers than non-GDM controls. Further research with larger sample sizes and complete pregnancy coverage is needed to explore the clinical utility such as screening and predictive values of CGM for GDM.

Hyperglycemia during pregnancy without a history of diabetes is known as gestational diabetes mellitus (GDM), with worldwide prevalence of 5–10% before 2010 (1–3). However, the prevalence has greatly increased after the new evidence-based guidelines from the International Association of Diabetes and Pregnancy Groups (IADPSG) were published and officially adopted in 2013 (1). The criteria was developed from the Hyperglycemia and Adverse Pregnancy Outcomes (HAPO) study (2) which showed that glucose levels correlate with maternal and fetal outcomes in a linear trend (3). Since the new criteria lowered the fasting glucose threshold significantly compared to other international guidelines [e.g., 5.1 mmol/L (92 mg/dL) vs. World Health Organization (WHO) 1999 7.0 mmol/L (126 mg/dL) (4) vs. National Institute for Clinical Evidence (NICE) 6.1 mmol/L (110 mg/dL)] (5), the GDM prevalence in 2015 increased by 2–3 folds to 25.1% in Singapore, 18% in Brazil, and 45.3% in United Arab Emirates (4).

Once a pregnant mother is diagnosed with GDM, she will be treated with either diet, medication (i.e., insulin), or both. In addition, she will be required to monitor her own glucose level closely using self-monitoring of blood glucose (SMBG) that involves finger pricking up to seven times daily. However, SMBG provides an incomplete picture of the daily glucose profile due to long intervals between finger pricking, and inaccurate self-reported measures which heavily rely on patients' compliance (6). Furthermore, reported barriers of using SMBG include stigma of testing in public places, pain, inconvenience, (7) and patient anxiety (8). Thus, there is a growing need for newer devices that could provide more frequent glucose measurements, improve patient compliance, and increase accuracy in reported data.

In the past decade, continuous glucose monitoring (CGM)—a new device that uses a subcutaneous sensor to measure interstitial fluid glucose levels—has been developed and advanced greatly in terms of clinical application (9). It offers a continuous measure of glucose profile and has been proven to show comparable accuracy compared with SMBG (6). Furthermore, CGM is more promising in clinical practice than SMBG in terms of a higher frequency of testing, trends in glucose values, alarms for dysglycemia (especially hypoglycemia) detection, therapy optimization, and identification of glucose fluctuations (10). With the wide adoption in clinical practice, CGM use has been shown to improve HbA1c and reduce glucose variability in patients with Type 1 Diabetes (T1D) (10) and is better for treatment monitoring than SMBG use in patients with Type 2 Diabetes (T2D) (11). Although CGM has been used successfully in T1D and T2D patients, the effectiveness of CGM in improving pregnancy outcomes complicated by GDM is still understudied.

In this review, we aimed to summarize and evaluate the use of CGM technology in pregnancies complicated by GDM, especially pertaining to feasibility, acceptability and efficacy (i.e., improvement in clinical outcomes and treatment effect). We hypothesized that there is strong evidence on the potential values of CGM in detecting more complete gestational glucose profiling, more episodes of dysglycemia, and in improving pregnancy outcomes and glycemic control among pregnant women with GDM.

## Methods

**Figure 1:**
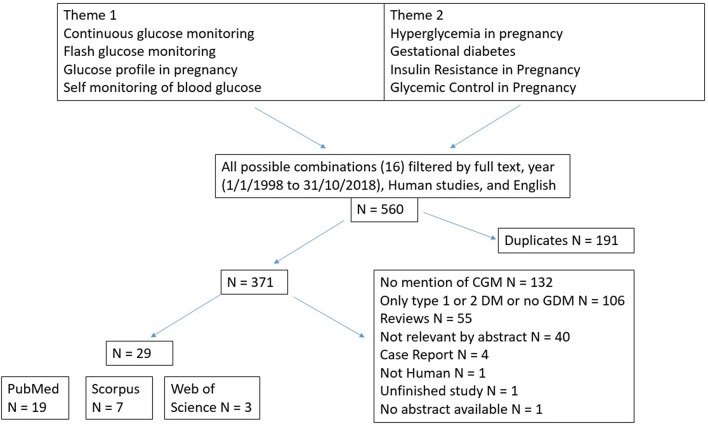
Literature search flowchart.

We conducted a broad search (conducted by QY and verified by L-JL) on PubMed, Scopus, and Web of Science using possible combination of terms from two themes featuring CGM and GDM, as shown in Figure 1. We first filtered full-text articles published between Jan 1st, 1998 and Dec 31st, 2018 that were mainly human studies and written in English. With these inclusion criteria, 560 articles remained. Second, we excluded duplicate articles (*n* = 191). Third, we removed articles without relevant titles (*n* = 132). Fourth, we excluded peer-reviews (*n* = 55) and lastly, we excluded articles without relevant abstracts (*n* = 105). We included a total of 29 articles in this review.

## Results

Table 1 shows the characteristics of the 29 studies included, of which 3 were randomized controlled trials (RCTs), one was a randomized crossover trial, and the remaining 25 were prospective observational cohorts with a small to medium number of subjects (*n* = 8–340). The majority of studies (*n* = 20) used Minimed (Medtronic, Dublin, Ireland) with 72 h monitoring. Other studies applied devices including GlucoDay S (GDS) (A.Menarini Diagnostics Ltd, Florence, Italy) (*n* = 2), Guardian (Medtronic, Dublin, Ireland) (*n* = 1), Seven and Seven Plus (Dexcom, San Diego, USA) (*n* = 1), iPro2 (Medtronic, Dublin, Ireland) (*n* = 4) and Freestyle Libre Flash Glucose Monitoring (FGM) (Abott, Illinois, USA) (*n* = 1).

**Table 1:** Characteristics of included studies.

	**References, Country**	**Population (GA in weeks)**	**Device and usage**	**Study design**	**Exposure**	**Outcomes**	**Limitations**
1	Yogev et al. (12) UK	*N* = 2 (24–32 weeks) 2GDM	72-h MiniMed, Medtronic Twice: 1x baseline, 1x post-treatment (2–4 weeks later)	Prospective observational study	CGM vs. SBGM use	Differences in glucose levels and insulin regimen	1. Under-controlled study 2. Small number of patients, 3. No clinical difference in perinatal outcome between CGM and SBGM due to lack of power
2	Chen et al. (13) Isreal and USA	*N* = 57 (32–36 weeks) Isreal: 47 GDM 23 diet only 24 diet and insulin (24–32) USA: 10 GDM	72-h MiniMed Medtronic Once	Prospective observational study	CGM vs. SBGM use	Daily glycemic profile	1. Non-standardized data analyses between two study sites
3	Ben-Haroush et al. (14) USA	*N* = 45 (25–32 wks avg) 45 GDM 26 by diet 19 by insulin	72 h MiniMed Medtronic Once	Prospective observational study	CGM vs. SMBG use	Peak postprandial glucose levels	1. Small sample size 2. No data on association between postprandial glucose levels and pregnancy outcomes
4	Buhling et al. (15) Germany	*N* = 64 (31–34 weeks) 8 non-pregnant NGT 56 pregnant women: 17 diet treated GDM (2 or 3 abnormal value on 75 g OGTT) 15 with IGT (1 abnormal value on 75 g OGTT) 24 NDP women (31–34)	72-h MiniMed Medtronic Once	Prospective observational study	CGM vs. SBGM use	Glucose profile	1. Small number of patients 2. No assessment of the clinical significance of hyperglycemic intervals
5	Yogev et al. (16) USA	*N* = 117 (32–33 weeks) 82 GDM: 27 diet only 30 on insulin 25 treated with glyburide 35 NDP women	72-h MiniMed Medtronic Once	Prospective observational study	CGM vs. SBGM	Hypoglycemic episodes	1. Did not study perinatal or maternal outcomes 2. No significant hypoglycemic events were identified in any patients
6	Buhling et al. (17) Germany	*N* = 49 (24–37 weeks) 36 NDP 13 GDM	72-h MiniMed Medtronic Once	Prospective observational study	CGM vs. SBGM use and diet	Postprandial glucose time to peak Postprandial glucose values	1. Unable to detect clinical outcome differences due to lack of power from small number of study subjects
7	Cypryk et al. (12) Poland	*N* = 19 (>28 wks) 12 GDM: 7 diet only 5 diet and insulin 7 NDP	72-h MiniMed Medtronic Once	Prospective observational study	CGM vs. SBGM	Glycemic control	1. Small number of study subjects 2. No fetal/obstetric outcomes measured
8	McLachlan et al. (18) Australia	*N* = 36 (10–34 weeks) 36 GDM	72-h MiniMed Medtronic Once	Prospective observational study	CGM vs. SBGM use	Clinical usefulness Patient assessment of usefulness Accuracy of CGMS	1. No blinded third party for assessing CGMS results 2. No statistical analysis of data 3. Not all target women agreed to participate which could lead to overestimation of usefulness of CGMS
9	Kestila et al. (19) Finland	*N* = 73 (>28 weeks) All with GDM 36 with GDM using CGM 37 with GDM using SMBG	Average 47.4-hr MiniMed, Medtronic Once	Prospective observational study	CGM vs. SBGM use	Determination of medical intervention	1. Study was not powered to detect differences in obstetrical outcome such as macrosomia. 2. Some mothers were not treated with antihyperglycemic medication even though indicated based on CGMS values
10	Seshiah et al. (20) India	*N* = 24 (22–30 weeks) 12 with GDM 12 NDP	72 h MiniMed Medtronic Once	Prospective observational study	CGM use	Postprandial time to peak	1. Only 3 SMBG measurements per day 2. Small sample size
11	Dalfra et al. (3) Italy	*N* = 48 (GA not specified) 31 with GDM (Carpenter and Coustan criteria^1^), 17 NDP	48-h GlucoDay S (GDS) Menarini Diagnostics Twice: 1 in 2nd trimester 1 in 3^rd^ trimester	Prospective observational study	CGM vs. SBGM use	Relationship between glycemic profiles and fetal growth	1. Small number of patients 2. BMI in women with diabetes was significantly higher before pregnancy compared to controls
12	Mazze et al. (21) USA	*N* = 82 (>27 weeks) 51 NDP 25 GDM 6 Pre-GD 21 non-pregnant NGT	72-h Guardian, Medtronic Once during 3rd trimester	Prospective, observational study	CGM vs. SBGM use in glyburide vs. insulin vs. diet	Diurnal glucose patterns of women	1. Only 3 days of testing for each sensor 2. Women were not randomly selected
13	Colatrella et al. (22) Italy	*N* = 33 (postpartum) 18 with GDM 15 NDP	72-h MiniMed, Medtronic Once	Prospective observational study	Suckling effect, CGM vs. SBGM use	Glucose profiles	1. Significant difference in BMI between groups 2. It cannot be ruled out that differences in glycemic profiles between groups could be due to diet
14	Dalfra et al. (23) Italy	*N* = 30 20 with GDM 10 NDP	48 h GlucoDay Once each trimester	Prospective observational study	CGM use	Glycemic variabilityHbA1c	1. Small sample size 2. Short monitoring period 3. Multiple recruitment centers with subsequent pooling of study population
15	Su et al. (24) China	*N* = 70 (25 weeks) 30 GDM pregnant women 20 NDP 20 non-pregnant NGT	72-h MiniMed, Medtronic Once	Prospective observational study	CGM vs. SBGM use	Glycemic variability and its association with B cell function	1. Study was not powered to detect associations between glycemic variability and pregnancy outcomes
16	Hernandez et al. (25) USA	*N* = 16 (31 weeks avg) All with GDM	72 h MiniMed Medtronic Twice	Randomized crossover study	Higher vs. lower carbohydrate diets	Postprandial glucose levels and insulin levels	1. Small study sample 2. Short duration 3. Highly controlled diet exposure
17	Yu et al. (26) China	*N* = 340 (26 weeks) 190 GDM with SMBG 150 GDM with SMBG and CGM	72-h MiniMed, Medtronic In SMBG group, twice (1st and 5th week of study) In SMBG and CGM group, every 2-4 weeks from study start to delivery	Prospective observational study	CGM vs. SBGM use, insulin vs. diet	Maternal complications: PE, miscarriage, IUFD, cesarean delivery. Neonatal outcomes: GA, preterm birth, BW, BW percentile, neonatal complications	1. Sample size inadequate for substantial positive cases of neonatal complications 2. In routine care group CGM data was analyzed in relation to pregnancy outcomes even though they were obtained only in 1st and 5th week of study
18	Kusunoki et al. (27) Japan	*N* = 22 (13 weeks) All with GDM	48-h MiniMed, Medtronic Once during the first 3 weeks of GDM diagnosis	Prospective observational study	CGM vs. SMBG use	Postprandial hyperglycemia HbA1c	1. No controls of healthy pregnant women
19	Sung et al. (24) US	*N* = 53 (24–28 weeks) NDP 2-step ACOG 9 failed 50 g GCT and screened with 75 g OGTT Diagnosed with GDM	6–7 days Seven and Seven Plus, Dexcom Avg use 4.8 days	Prospective observational study	CGM vs. SMBG use, food diary effect	Primary outcome: BW percentile Secondary outcomes: unplanned operative delivery and macrosomia	1. The GDM diagnostic criteria was changed midway through study 2. Data was collected only during 24–28 weeks gestation 3. Sample size was underpowered to detect differences in secondary outcomes
20	Wang et al. (28) China	*N* = 96 48 NDP with previous GDM 48 NDP w/out previous GDM	72 h MiniMed Medtronic Once	Prospective observational study	CGM use	Glycemic variability	1. Sample size was underpowered to detect differences in subgroups 2. Factors such as physical activating and emotional stress could not be controlled and could affect glycemic variability
21	Alfadhli et al. (29) Saudi Arabia	*N* = 130 (26 weeks avg) 62 with GDM and SMBG 68 with GDM and CGM	3–7 days Minimed Medtronic Once	Prospective open label randomized controlled study	CGM vs. SMBG use	Maternal glycemic control Pregnancy outcomes Glucose variability	1. Single use of CGM 2. Small sample size
22	Carreiro et al. (30) Brazil	*N* = 50 (27–36 weeks) 36 with GDM 14 non-pregnant BMI matched NGT	72-h Minimed, Medtronic Once	Prospective observational study	CGM vs. SMBG use	Glucose profiles Effects of dietary counseling on glucose profiles	1. Pooled glucose profiles is one summary point rather than all measurements. 2. No analysis on perinatal outcomes 3. No evaluation of the same patients before and after dietary counseling with CGM
23	Wei et al. (31) China	*N* = 120 (24–36 weeks) 58 with GDM in CGM 62 with GDM in SMBG	48–72-h Gold MiniMed, Medtronic Once	Prospective, observational, open-label randomized controlled trial	CGM vs. SMBG use, diet vs. insulin	Maternal: GWG, cesarean section Neonatal: BW Apgar score at 5 min HbA1c levels Glucose variability	1. Small sample size with no significant differences in outcomes 2. Education management was not blinded (Hawthorne effect)
24	Naik et al. (32) India	*N* = 30 (24–36 weeks) 20 with GDM 10 NDP	72-h MiniMed Medtronic Once	Prospective observational study	CGM vs. SMBG, Medical nutrition intervention vs. insulin	Masked hypoglycemia (interstitial glucose levels <2.7 mmol/L [48.6 mg/dL] for >30 min without symptoms detected by CGM)	1. More women in CGM group underwent cesarean section but most of them were elective 2. Small sample size
25	Panyakat et al. (33) Thailand	*N* = 55 (28–32 weeks) All with GDM	72 h iPro2 Medtronic Once	Prospective observational study	CGM use	Glycemic variability Pregnancy outcomes	1. Low incidence of perinatal outcomes 2. Study conducted in third trimester only
26	Paramasivam et al. (34) Malaysia	*N* = 50 (28, 32, 36 weeks) All insulin-treated GDM: 25 with CGM 25 with SMBG	7 days iPro2 Medtronic Three times	Prospective randomized open label controlled trial	CGM vs. SMBG use	HbA1c	1. Small sample size 2. Unblinded participants and practitioners
27	Pustozerov et al. (35) Russia	*N* = 62 (31 weeks avg) 49 with GDM 13 NDP	7 days iPro2 Medtronic	Prospective observational trial	Mobile app, CGM use vs. SMBG	Postprandial peak glucose levels Postprandial time to peak FBG	1. Self-reported food intake 2. Small sample size 3. Accuracy of prediction models has not been proven
28	Scott et al. (36) UK and Austria	*N* = 39 (26.6 weeks avg) 39 with GDM	Up to 14 days FreeStyle Libre FGM system (Abbott Diabetes Care) Once	Prospective observational trial	CGM vs. SMBG	Accuracy User acceptability Safety evaluation	1. Sample size not powered to detect accuracy between subgroups 2. Short term study
29	Voormolen et al. (37) Holland	*N* = 109 (<30 weeks) 109 with GDM Among them, 147 using CGM 153 using standard treatment	5–7 day iPRO2 retrospective CGM, Medtronic Once in every 6 weeks	Open label, multicenter, randomized controlled trial	CGMS vs. SMBG use	Primary outcome: macrosomia, Secondary outcomes: BW, neonatal and maternal morbidity HbA1cGlucose variability	1. Enrollment took place over more than 4 years 2. High number of patients refused continued use of CGM 3. Cannot compare CGM related outcomes b/c not blinded

1 * Carpenter and Coustan criteria: 3 h OGTT with fasting ≥ 95 mg/dL [5.4 mmol/L], 1 h ≥ 180 mg/dL [10 mmol/L], 2 h ≥ 155 mg/dL [8.6 mmol/L], or 3 h ≥ 140 mg/dL [7.8 mmol/L]*. *GA, Gestational age; CGM, Continuous glucose monitoring; SMBG, Self-monitoring of blood glucose; GDM, Gestational diabetes; T1D, Type 1 diabetes; IGT, Impaired glucose tolerance; NDP, Non-diabetic pregnancy; T2D, Type 2 diabetes; NGT, Normal glucose tolerance; OGTT, Oral glucose tolerance test; BMI, Body mass index; PE, Preeclampsia; GA, Gestational age; BW, Birth weight; GWG, Gestational weight gain; FBG, Fasting blood glucose*.

We summarize the major findings categorized below:

### CGM vs. SMBG in Terms of Feasibility and Acceptability (Table 2)

Two studies which focused on user acceptability found that CGM use was generally well-tolerated by patients. McLachlan et al. demonstrated that CGM was able to provide an additional 62% of information that is missing in patients' self-recorded glucose diaries (18). Most patients felt that CGM is easy to use (44 out of 48, 92%), beneficial for self-glycemic control (43 out of 48, 90%), and that its use outweighed its inconvenience (37 out of 48, 77%) (18). Likewise, Scott et al. demonstrated that CGM had acceptable accuracy (88.1% within Zone A of Consensus Error Grid) with only 11.8% mean difference with SMBG (36).

**Table 2:** Other outcomes of articles.

	**References, Country**	**Other outcomes**
1	McLachlan et al. (18) Australia	1. Positive user feedback 2. CGM vs. SMBG: ↑ Information gathering
2	Colatrella et al. (22) Italy	Suckling did not affect blood glucose profiles significantly.
3	Wang et al. (28) China	Previous GDM vs. w/out previous GDM: ↓ Integrated B cell function index 90% acceptability
4	Scott et al. (36) UK and Austria	CGM vs. SMBG: Acceptable accuracy with 11.8% absolute relative difference High user acceptabilityNo AE

*CGM, Continuous glucose monitoring; GDM, Gestational diabetes; SMBG, Self-monitoring of blood glucose*.

### CGM vs. SMBG and Risk of Adverse Pregnancy Outcomes (Table 3)

Two RCTs and 1 prospective cohort showed no differences in any maternal and fetal outcomes between CGM use and SMBG use among GDM pregnancies, while 3 other studies, including 1 RCT and 2 prospective cohorts, reported otherwise.

**Table 3:** Major findings of articles with focus on pregnancy outcomes.

	**References, Country**	**Pregnancy outcomes**	
		**Maternal**	**Fetal**
1	Kestila et al. (19) Finland	CGM vs. SMBG: HDP: Nil C-section: Nil GA at delivery: Nil	CGM vs. SMBG: BW: Nil Neonatal hypoglycemia: Nil
2	Yu et al. (26) China	CGM vs. SMBG: ↓ PE ↓ PTD ↓ C-section	CGM vs. SMBG: ↓ BW ↓ Macrosomia ↓ LGA ↓ Neonatal complications (hypoglycemia, hyperbilirubinemia, RDS)
3	Sung et al. (38) USA		Hyperglycemia and BW %tile: Positive correlation
4	Alfadhli et al. (29) Saudi Arabia	CGM vs. SMBG: C-section:Nil GA: Nil	CGM vs. SMBG: BW: Nil Macrosomia: Nil Neonatal hypoglycemia: Nil
5	Wei et al. (31) China	CGM vs. SMBG: C-section: Nil	CGM vs. SMBG: BW: Nil Macrosomia: Nil LGA: Nil SGA: Nil Neonatal hypoglycemia: Nil APGAR 5 min: Nil
6	Voormolen et al. (37) Holland	CGM vs. SMBG: HDP: Nil HELLP: Nil ↓ incidence of PE	CGM vs. SMBG: BW: Nil Macrosomia: Nil LGA: Nil SGA: Nil

*GA, Gestational age; HDP, Hypertensive disorders of pregnancy; c-section, Cesarean section; BW, Birth weight; PCOS, Polycystic ovarian syndrome; LGA, Large for gestational age; SGA, Small for gestational age; PE, Pre-eclampsia; PTD, Preterm delivery*.

#### Maternal Outcomes

Wei et al. randomly assigned 120 pregnant women with GDM to either CGM (*n* = 58) or SMBG (*n* = 62) (31). CGM monitoring was done once for 48–72 h via MiniMed device (Medtronic, Dublin, Ireland) while traditional treatment involved SMBG (4 times daily) and HbA1c levels (every 4 weeks) (31). In this RCT, Wei et al. found no significant differences in cesarean section rates or HbA1c levels between CGM and SMBG users (31). Alfadhli et al. recruited 130 patients with GDM (62 SMBG, 68 CGM) and found no significant differences in cesarean section rates between the groups (29).

In addition, one prospective observational study also found no significant differences in maternal outcomes between CGM and SMBG groups. Kestila et al. enrolled 73 women with GDM and assigned 36 to CGM and 37 to SMBG (19). No differences were found in terms of frequency of pre-eclampsia, pregnancy-induced hypertension, maternal lacerations, and cesarean section rate between the 2 groups (19).

On the contrary, 2 studies found significant differences. Voormolen et al. randomized 109 women with GDM to CGM vs. standard treatment (SMBG 4–8 times daily and HbA1c levels every 4 weeks) (37). Compared with SMBG users, CGM users had a significantly lower incidence of pre-eclampsia [Relative Risk (RR) 0.3; 95%Cl: 0.12–0.8] (37). Yu et al. recruited 340 women with GDM and assigned 190 to routine care (SMBG 7 times daily) and the other 150–72 h CGM (26). Compared with SMBG, CGM users tended to have lower incidence of pre-eclampsia [5 out of 150 (3.3%) vs. 19 out of 190 (10%), *P* = 0.019], primary cesarean section [51 out of 150 (34.0%) vs. 88 out of 190 (46.3%), *P* = 0.028], and premature delivery [7 out of 150 (4.7%) vs. 22 out of 190 (11.6%), *P* = 0.024] (26).

#### Fetal Outcomes

The same studies also compared fetal outcomes between CGM and SMBG groups and all three RCTs found no significant differences (19, 29, 31, 37). Only one study by Yu et al. reported better fetal outcomes in the CGM group compared to SMBG group, in terms of BW (3,138 vs. 3,345 g, *P* < 0.001), numbers of macrosomia [6 out of 150 (4%) vs. 20 out of 190 (10.5%), *P* = 0.025], LGA [20 out of 150 (13.3%) vs. 48 out of 190 (25.3%), *P* = 0.01], neonatal hypoglycemia [8 out of 150 (5.3%) vs. 26 out of 190 (13.7%), *P* = 0.011], neonatal hyperbilirubinemia [4 out of 150 (2.7%) vs. 18 out of 190 (9.5%), *P* = 0.012], and neonatal respiratory distress syndrome (RDS) [2 out of 150 (1.3%) vs. 11 out of 190 (5.8%), *P* = 0.034] (26).

### CGM vs. SMBG and Detection of Dysglycemia (Table 4)

Two prospective observational studies that compared CGM vs. SMBG to detect dysglycemia concluded that CGM detected more hypoglycemia and hyperglycemia incidents. Chen et al. conducted a study in Israel (*n* = 47) and USA (*n* = 10) on glucose profiling between CGM and SMBG in women with GDM (13), and found that CGM detected hyperglycemic and hypoglycemic events better than SMBG (13). The other study by McLachlan et al. studied the clinical usefulness of CGM in 36 patients with GDM and had similar conclusions (18). They found that CGM detected postprandial hyperglycemia that was underestimated by SMBG (18).

**Table 4:** Major findings of articles focused on dysglycemia and glycemic profiling.

	**References, Country**	**Dysglycemia detection CGM vs. SMBG**	**Glycemic profiling**
1	Chen et al. (13) Isreal and USA	↑ Hypoglycemia ↑ Hyperglycemia	Hyperglycemia and Hba1c: Nil
2	Ben-Haroush et al. (14)USA		T1D vs. GDM: ↑ Variation in postprandial glucose time to peak ↓ Time interval to pre-prandial glucose value
3	Buhling et al. (15) Germany		GDM vs. NDP: ↑ Time to peak ↑ Hyperglycemia
4	Yogev et al. (16) USA		GDM vs. NDP: ↑ Hypoglycemia events
5	Buhling et al. (17) Germany		GDM vs. NDP: Postprandial glucose time to peak: Nil Postprandial glucose values: Nil
6	Cypryk et al. (12) Poland		GDM vs. NDP: Glycemic variability: Nil
7	McLachlan et al. (18)Australia	↑ Hyperglycemia	
8	Seshiah et al. (20) India		GDM vs. NDP: ↑ Postprandial glucose levels
9	Dalfra et al. (3) Italy		GDM w/insulin vs. GDM w/dietary restriction: ↑ Glycemic variability
10	Mazze et al. (21) USA		GDM vs. NDP: ↑ Glycemic variability
11	Colatrella et al. (22) Italy		GDM vs. NDP: ↑ Postprandial time to peak ↑ Postprandial levels
12	Dalfra et al. (23) Italy		GDM vs. NDP: ↑ Glycemic variability
13	Su et al. (24) China		GDM vs. NDP: ↑ Glycemic variability
14	Kusunoki et al. (27) Japan		Postprandial glucose levels and HbA1c: Positive correlation
15	Wang et al. (28) China		pGDM vs. w/out pGDM: ↑ Glycemic variability
16	Alfadhli et al. (29) Saudi Arabia		CGM vs. SMBG Glycemic variability improved
17	Carreiro et al. (30) Brazil		GDM vs. NDP: ↑ Variability ↑ Postprandial levels
18	Naik et al. (32) India		GDM vs. NDP: ↑ Postprandial levels
19	Panyakat et al. (33) Thailand		CGM: Glycemic variability and pregnancy outcomes associations: nil
20	Pustozerov et al. (35)Russia		GDM vs. NDP: ↑ Postprandial levels ↑ Fasting glucose

*CGM, Continuous glucose monitoring; SMBG, Self-monitoring of blood glucose; GDM, Gestational diabetes; NDP, Non-diabetic pregnancy; T1D, type 1 diabetes*.

### GDM vs. NDP in Terms of CGM-Derived Glycemic Profiling (Table 4)

#### Average Daily Glucose Levels

As expected, all three studies focusing on glucose profiling found that women with GDM had higher average glucose levels and that CGM was better than SMBG in detecting subtle glucose changes. Yogev et al. enrolled 117 patients (82 GDM vs. 35 NDP) and identified more asymptomatic hypoglycemic episodes (i.e., no dizziness or sweating due to hypoglycemia) by using CGM in the GDM group compared to the controls (25 vs. 0, *P* < 0.001) (16). Buhling et al. found similar results when they enrolled 8 non-pregnant healthy women and 56 pregnant women (17 diet-treated GDM, 15 impaired glucose tolerance (IGT) and 24 NDP) (15). The duration of hyperglycemia above 6.7 mmol/L [120 mg/dL] was higher in those with GDM or IGT than NDP (190 vs. 381.8 vs. 138 min; *P* < 0.05) (15). Colatrella et al. focused on glucose profiles during breastfeeding in two different groups: 18 women with GDM during pregnancy that resolved postpartum and 15 non-diabetic healthy pregnant controls (22). Glycemic levels were significantly higher in those who had GDM than NDP [5.6 mmol/L (101.4 mg/dL) vs. 4.9 mmol/L (87.5 mg/dL), *P* = 0.002] (22).

#### Postprandial Glucose Peak Level, Time to Peak, and Time to Resume to Baseline

The majority of research (4 out of 5, 80%) showed that patients with GDM had higher postprandial glucose levels. Naik et al. enrolled 30 women (20 GDM vs. 10 NDP) and found 1 h postprandial glucose levels were higher in women with GDM than NDP [7.21 mmol/L (130 mg/dL) vs. 5.98 mmol/L (108 mg/dL) *P* = 0.01] (32). Pustozerov et al. recruited 62 patients (49 GDM vs. 13 NDP) and demonstrated higher postprandial peak levels in patients with GDM than NDP [6.6 mmol/L (119 mg/dL) vs. 6.5 mmol/L (117 mg/dL), *P* = 0.02] (35). Carreiro et al. enrolled 50 patients (36 with GDM, 14 BMI-matched NDP) and found that at both breakfast and dinner times, postprandial glucose levels were consistently higher in the GDM group compared to NDP in both 1- and 2-h time points (i.e., 1 h after breakfast: 6.5 mmol/L [118 mg/dL] vs. 5.5 mmol/L [100 mg/dL]; 1 h after dinner: 6 mmol/L [109 mg/dL] vs. 5.2 mmol/L [94 mg/dL]; 2 h after breakfast: 5.8 mmol/L [105 mg/dL] vs. 4.9 mmol/L [89 mg/dL], 2 h after dinner: 5.8 mmol/L [105 mg/dL] vs. 5.2 mmol/L [93 mg/dL], *P* < 0.05 for all) (30). Finally, Seshiah et al. enrolled 24 women (12 GDM vs. 12 NDP) and found higher glucose levels 1 h post-meal in women with GDM than NDP, either using CGM [6.8 mmol/L (122.22 mg/dL) vs. 5.7 mmol/L (102.68 mg/dL), *P* < 0.05] or SMBG [7.2 mmol/L (129.9 mg/dL) vs. 6.5 mmol/L (117.5 mg/dL), *P* < 0.05] (20).

In terms of postprandial time to peak, there was no consensus among researchers. Buhling et al. conducted two studies focused on postprandial time to peak. Initially, they found that patients with GDM had longer time to peak compared to patients with NDP (54 vs. 47 min, *P* = 0.008) (15). Subsequently, they enrolled 49 women (13 GDM vs. 36 NDP) but found no significant differences in postprandial time to peak between NDP and diabetic groups (74 vs. 82 min, *P* > 0.05) (17). Carreiro et al. found similar results to Buhling's first study in that postprandial time to peak was longer in the GDM group than NDP group (56–70 min vs. 33 min, *P* < 0.02) at breakfast time (30).

For postprandial time to resume, only one study by Ben-Haroush et al. enrolled 45 women with GDM and found that women with diet-treated GDM took a longer time to return to pre-prandial glucose levels than those with insulin-treated GDM (134 vs. 111 min, *P* = 0.022) (14).

#### Glycemic Variability

Glycemic variability was consistently found to be increased in patients with GDM compared to NDP. Mazze et al. enrolled 76 patients (51 NDP, 25 GDM) and found significantly higher glucose variability in the GDM group compared to NDP [Interquartile range (IQR): 1.94 mmol/L (35 mg/dL) vs. 1.3 mmol/L (23 mg/dL), *P* < 0.0001] (21). Su et al. enrolled 70 women (30 GDM, 20 NDP, 20 non-pregnant healthy controls) and found similar results: mean amplitude of glucose excursions (MAGE) values were higher in the GDM group compared to both NDP and non-pregnant healthy controls [3.5 mmol/L (63 mg/dL) vs. 2.3 mmol/L (41 mg/dL) vs. 1.7 mmol/L (30.6 mg/dL); *P* < 0.01] (24). Other parameters of glycemic variability with significant differences between GDM and NDP included mean of daily differences (MODD) [1.6 mmol/L (29 mg/dL) vs. 1.2 mmol/L (21.6 mg/dL) vs. 1.0 mmol/L (18 mg/dL) *P* < 0.05], standard deviation of blood glucose (SDBG) [1.5 mmol/L (27 mg/dL) vs. 1.0 mmol/L (18 mg/dL) vs. 0.9 mmol/L (16 mg/dL), *P* < 0.01], and mean of continuous 24 h blood glucose (MBG) [8.6 mmol/L (155 mg/dL) vs. 6.2 mmol/L (112 mg/dL) vs. 4.8 mmol/L (86 mg/dL), *P* < 0.01] (24). There were two studies conducted by Dalfra et al. One focused on treatment differences in patients with GDM (insulin vs. diet), and they found patients on insulin had significantly higher glycemic variability compared to those on dietary restriction [MAGE: 3.5 mmol/L (63.3 mg/dL) vs. 2.1 mmol/L (38 mg/dL); *P* = 0.012] (3). The other study compared 30 patients (20 GDM vs. 10 NDP) and found that GDM had significant correlations between indicators of glycemic variability (MAGE and IQR *r* = 0.84, *P* < 0.001; MAGE and continuous overlapping net glycemic action (CONGA) *r* = 0.54, *P* = 0.03) (23). Only one study by Alfadhli et al. compared glucose differences between day 1 and day 4 use of CGM device among GDM mothers (29). They found significant reduction of glucose levels after a 4-day's use of CGM [MBG: 1.0 mmol/L (18 mg/dL), *P* = 0.016; SDBG: 0.25 mmol/L (4.5 mg/dL), *P* = 0.034] (29).

Interestingly, there was one study which compared mothers of NDP with and without history of GDM. Wang et al. recruited 96 women (48 NDP with prior GDM and 48 without) and found that those with prior GDM had higher MBG [6.5 mmol/L (117 mg/dL) vs. 5.9 mmol/L (106 mg/dL), *P* = 0.004], SDBG [1.3 mmol/L (23 mg/dL) vs. 0.9 mmol/L (16 mg/dL), *P* < 0.001], MODD [1.4 mmol/L (25 mg/dL) vs. 1.1 mmol/L (19.8 mg/dL), *P* = 0.002], MAGE [2.7 mmol/L (48.6 mg/dL) vs. 1.8 mmol/L (32.4 mg/dL), *P* < 0.001], and AUC [26.8 mmol/L (483 mg/dL) vs. 19.2 mmol/L (346 mg/dL) per hour, *P* < 0.001] compared to NDP without prior GDM (28).

However, one study did report contrasting results relating to glycemic variability from the findings above. Cypryk et al. enrolled 19 pregnant women (12 GDM, 7 NDP) and found no significant differences between groups for mean 24 h glycaemia, mean glucose level during the night, and duration of glycaemia below 3.3 mmol/L [60 mg/dL] or above 6.7 mmol/L [120 mg/dL], regardless of whether CGM or SMBG was used to measure the parameters (12).

### CGM Use in Clinical/Intervention Utility (Table 5)

#### CGM in Altering Treatment Effect

All four studies focused on using CGM to make medication adjustments in patients with GDM found that CGM led to more treatment changes. Yogev et al. recruited 2 GDM patients and CGM was worn twice (once at baseline and once at 4 weeks after treatment) (39). After adjusting for their insulin regimens, there was a significant decrease in the total time of undetected hyperglycemia (152 vs. 89 min/day, *P* < 0.03) and in the 24-h mean glucose levels [6.5 mmol/L (117 mg/dL) vs. 5.6 mmol/L (101 mg/dL), *P* < 0.02] at post-treatment evaluation (39). Kestila et al. found similar results in their study of 73 patients with GDM (36 CGM, 37 SMBG) (19). Those in the CGM group had more patients treated with anti-hyperglycemic medication (31 vs. 8%, *P* = 0.01) (19). Wei et al. compared CGM use (*n* = 58) to SMBG use (*n* = 62) in 120 women with GDM and found that insulin was more commonly used in the CGM group (31.3 vs. 12.7%, *P* = 0.02), although there was no significant difference in the required dose (31). Yu et al. also found that more women in the CGM group received insulin vs. SMBG [41 out of 150 (27.3%) vs. 23 out of 190 (12.1%), *P* < 0.001] (26).

**Table 5:** Major findings of articles with focus on clinical and intervention utility.

		**Clinical/Intervention utility**	
	**References, Country**	**Treatment effect**	**Maternal GWG**
1	Yogev et al. (12) UK	↑ Medical monitoring and treatment adjustment	
2	Kestila et al. (19) Finland	↑ Medical monitoring and treatment adjustment	
3	Hernandez et al. (25) USA	↑Complex-carbohydrate and ↓ fat diet maintained glycaemia below guidelines	
4	Wei et al. (31) China	↑ Medical Monitoring and treatment adjustment	CGM vs. SMBG: ↓
5	Panyakat et al. (33) Thailand		GWG and BW correlated

*GWG, Gestational weight gain; CGM, Continuous glucose monitoring; SMBG, Self-monitoring of blood glucose; BW, Birth weight*.

One trial conducted by Paramasivam et al. studied CGM vs. SMBG use in preventing HbA1c increases in 50 women with insulin-treated GDM (34). They found that HbA1c had a smaller increase in the CGM group (*P* = 0.024) and that mean HbA1c was lower in the CGM group at 37 weeks (*P* < 0.006) (34). The vast majority of patients in the CGM group also achieved an HbA1c <39 mmol/mol (5.8%) at 37 weeks (92 vs. 68%, *P* = 0.012) (34).

#### CGM Effect on Gestational Weight Gain (GWG)

Only one RCT studied GWG and found that CGM use lowered the amount of GWG compared to SMBG. Wei et al. recruited 120 women with GDM and randomized them to CGM (*n* = 58) or SMBG (*n* = 62), and found a significant difference in GWG (13.56 kg vs. 14.75 kg, *P* = 0.004) (31). A prospective observational study by Panyakat et al. did find a significant correlation between GWG and BW percentiles using CGM (*r* = 0.437, *P* = 0.002) (33).

### Other Implications of CGM Use

#### Postprandial Glucose and HbA1c Levels (Table 4)

Kusunoki et al. enrolled 22 patients with GDM and used CGM to find that postprandial glucose levels were positively correlated with HbA1c levels (*r* = 0.5, *P* = 0.03) and patients' BMI (*r* = 0.55, *P* = 0.01) (27).

#### Hyperglycemia and Birth Weight (Table 3)

Sung et al. recruited 53 healthy pregnant women and followed them with gestational diabetes screening (38). They found that the magnitude and duration of hyperglycemia (≥110 mg/dl) had a consistent positive correlation with BW (Area under the curve (AUC) 6.1–7.8 mmol/L [110–140 mg/dl]: all *r* = 0.29, all *P* < 0.05) (38).

#### Diet and Average Glycaemia (Table 5)

Hernandez et al. used CGM to monitor changes in glycaemia in a crossover study using two different diets: a conventional lower carbohydrate and higher fat diet vs. a higher-complex carbohydrate and lower fat diet (25). They found that although the higher-complex diet led to higher levels of average glucose levels, overall the diet still maintained glycaemia below the recommended guidelines (25).

#### Pregnancy Outcomes (Table 4)

A different study by Panyakat et al. which enrolled 55 women with GDM and investigated the associations between third trimester glucose variability parameters and pregnancy outcomes using CGM (33). They found no associations between the two in terms of LGA, BW, cesarean section rate, and neonatal complications (33).

## Discussion

In this systematic review with 29 online published original articles, CGM use has better user acceptability and feedback regarding satisfaction than SMBG use in daily glucose monitoring. Furthermore, most studies showed that CGM use in pregnant women with GDM was more effective in detecting hypoglycemia, hyperglycemia and increased glycemic variability than SMBG use in both GDM subjects and non-GDM subjects. As for treatment effect, CGM use led to more frequent use of insulin, better glycemic control, and reduced GWG in patients with GDM. However, most studies showed inconclusive results regarding CGM use in order to improve maternal and fetal outcomes.

Current research shows that CGM is able to provide more comprehensive glucose data and is convenient for the patient (40). Its clinical utility has been well demonstrated in patients with T1D and T2D by reducing risks of dysglycemia and improving their quality of life (41). However, several questions remain unanswered with regards to CGM use in patients with GDM: (1) Whether CGM can *detect early* glycemic variability for GDM diagnosis; (2) Whether CGM can *subsequently* moderate treatment strategies of GDM; (3) Whether CGM *can eventually* improve maternal and fetal outcomes. Due to these gaps in knowledge, CGM has yet to be adopted widely for use in pregnancies complicated by GDM.

In our review, there are consistent findings on the effect of CGM use on detection of dysglycemia (13, 18), higher glycemic variability (3, 21, 23, 24, 28, 29), higher postprandial glucose peaks in women with GDM (20, 30, 32, 35), and improved treatment effect (19, 25, 31, 34, 39). CGM use contributed positively to treatment effect as clinicians were more aware of patients' hyperglycemic and hypoglycemic episodes, so as to moderate the strategies of their medication (i.e., insulin) by altering dosage and frequency. In general, these findings suggest better clinical utility of CGM use in patients with GDM, compared with traditional SMBG use.

One major gap in clinical knowledge—whether CGM use improves maternal and fetal outcomes—has not been filled with plausible or convincing results. Past findings are still equivocal, as described above (26, 29, 31, 37). The null findings might be due to the lack of study power to detect pregnancy outcomes which are not highly prevalent in relatively small samples (42). Therefore, future studies with larger samples size, longer follow-up, and consistent study design are warranted to detect the practical effect of CGM on maternal and fetal outcomes.

There are a few other aspects of CGM which are worth exploring in future research, such as pregnant users' acceptability and effect on GWG and glycemic control. In our review, only two studies surveyed user acceptability and accuracy of CGM (18, 36), while several studies have been done in patients with T2D with high compliance rates (>90%) (43). Therefore, more should be done on pregnant women, especially on those with GDM, in order to implement universal application of CGM during pregnancy. Future research that tackles the current challenges and difficulties of CGM use in women with or without hyperglycemia during pregnancy could be beneficial for CGM user compliance and glucose control. In addition, one study in our review reported a beneficial effect of CGM on reducing GWG (31). It is worth further investigating whether such self-regulatory effect of CGM use (i.e., reduced GWG and lower glycemic levels during pregnancy) has an impact on improving pregnancy and fetal outcomes.

The strength of this literature review was the novelty of summarizing the clinical utility and treatment effects of CGM use on pregnancies complicated by GDM only, via a set of stringent selection criteria across three reputable medical databases. However, this review is not without limitations. First, there was a possibility of selection bias as we only included articles written in English with full text available and published in the past 2 decades. Second, when filtering articles by title, we might have excluded relevant articles that did not have the keywords (i.e., CGM, pregnancy, GDM) in their title.

In summary, there is sufficient evidence showing that CGM is effective at capturing gestational glucose profiles and improving treatment effect among pregnant women with GDM. The use of CGM provides good user acceptability, detects more dysglycemia than SMBG use, and detects higher glycemic variability in GDM pregnancies than normal pregnancies. Since CGM use is somewhat effective at improving GDM pregnancy outcomes, further research with larger sample sizes, better compliance, and longer monitoring times to detect these effects are warranted.

## Data Availability Statement

All datasets for this study are included in the manuscript and the supplementary files.

## Author Contributions

QY drafted the manuscript and conducted the literature search. IA edited the manuscript and partially conceived the research idea. KT edited the manuscript and provided funding for this research. L-JL verified the literature search, conceived the research idea, and edited the manuscript.

### Conflict of Interest

The authors declare that the research was conducted in the absence of any commercial or financial relationships that could be construed as a potential conflict of interest.

## Abbreviations

GDM: Gestational diabetes
SMBG: Self-monitoring of blood glucose
CGM: Continuous glucose monitoring
RCTs: Randomized controlled trials
BW: Birth weight
GA: Gestational age
GWG: Gestational weight gain
NDP: Non-diabetic pregnancies
IADPSG: Internal Association of Diabetes and Pregnancy Groups
HAPO: Hyperglycemia and adverse pregnancy outcome
WHO: World Health Organization
NICE: National Institute for Clinical Evidence
T1D: Type 1 Diabetes
T2D: Type 2 Diabetes
GDS: GlucoDay S
NICU: Neonatal intensive care unit
IGT: Impaired glucose tolerance
PCOS: Polycystic ovarian syndrome
BMI: Body Mass Index
IQR: Interquartile range
MAGE: Mean amplitude of glucose excursions
MODD: Mean of daily differences
SDBG: Standard deviation of blood glucose
MBG: Mean of continuous 24 h blood glucose.
